# Stroke rehabilitation research needs to be different to make a difference

**DOI:** 10.12688/f1000research.8722.1

**Published:** 2016-06-22

**Authors:** Cathy M. Stinear

**Affiliations:** 1 Department of Medicine, University of Auckland, Auckland, New Zealand

**Keywords:** Stroke, rehabilitation, intervention assessment

## Abstract

Stroke continues to be a major cause of adult disability. In contrast to progress in stroke prevention and acute medical management, there have been no major breakthroughs in rehabilitation therapies. Most stroke rehabilitation trials are conducted with patients at the chronic stage of recovery and this limits their translation to clinical practice. Encouragingly, several multi-centre rehabilitation trials, conducted during the first few weeks after stroke, have recently been reported; however, all were negative. There is a renewed focus on improving the quality of stroke rehabilitation research through greater harmonisation and standardisation of terminology, trial design, measures, and reporting. However, there is also a need for more pragmatic trials to test interventions in a way that assists their translation to clinical practice. Novel interventions with a strong mechanistic rationale need to be tested in both explanatory and pragmatic trials if we are to make a meaningful difference to stroke rehabilitation practice and outcomes.

## Background

Stroke is a leading cause of adult disability worldwide ^1^. Advances in stroke prevention have led to a decline in stroke incidence, particularly in developed countries ^2^. There have also been recent advances in acute stroke treatment with thrombolysis and clot retrieval ^3^. However, the number of people living with the consequences of stroke continues to rise ^2^. Stroke rehabilitation has steadily evolved with new service delivery models and a greater understanding of the importance of therapy intensity and task specificity. However, the search continues for new therapies that can be widely incorporated into routine clinical practice, despite more than 1000 randomised controlled trials (RCTs) having been conducted ^4^.

One factor that may limit the translational impact of stroke rehabilitation RCTs is their timing. It is important to conduct rehabilitation trials during the initial days and weeks after stroke because this is when spontaneous biological recovery (SBR) is taking place ^5^ and when rehabilitation is delivered in the ‘real world’. Testing an intervention at the time of its intended use is crucial for evaluating its efficacy as well as its feasibility in clinical practice. We have found that over half of motor rehabilitation RCTs are conducted with patients who are at least 6 months post-stroke, when rehabilitation services are no longer available to most patients ^6^. Only 5% of RCTs were of good quality and recruited all patients within 30 days of stroke. Of these, half were negative. Compared with negative trials, the positive trials were more likely to recruit fewer than 40 patients and have no follow-up measures. There is clearly a need for larger trials conducted early after stroke in the real-world clinical setting.

In the last 18 months, six multi-centre rehabilitation RCTs that recruited patients within the first 3 months after stroke have been reported. These trials are summarised below.

1. SIRRACT (Stroke Inpatient Rehabilitation Reinforcement of ACTivity) recruited 135 patients within 45 days after stroke at 16 sites over the course of 15 months ^7^. Participants were randomly assigned to either standardised verbal feedback about walking speed or augmented feedback based on activity graphs derived from wireless activity sensors. The primary outcomes were average daily time spent walking during inpatient rehabilitation and the fastest safe 15-metre walking speed at discharge from inpatient rehabilitation.

2. CIRCIT (Circuit class therapy or seven-day week therapy for Increasing Rehabilitation Intensity of Therapy after stroke) recruited 283 patients between 5 and 197 days (mean of 28 days) after stroke at five sites in 36 months ^8^. Participants were randomly assigned to usual care therapy 5 days per week, usual care therapy 7 days per week, or circuit class therapy 5 days per week. The primary outcome was the 6-minute walk test at 4 weeks post-randomisation.

3. AVERT (A Very Early Rehabilitation Trial) recruited 2104 patients within 24 hours of stroke symptom onset at 56 sites over the course of 100 months ^9^. Participants were randomly assigned to usual care or very early mobilisation, which required at least three additional out-of-bed sessions targeting standing and walking beginning within 24 hours of stroke. The primary outcome was a favourable outcome 3 months after stroke, defined as a modified Rankin Scale score of 0, 1, or 2.

4. A trial of acupuncture recruited 862 patients between 3 and 10 days after stroke at 40 sites in 35 months ^10^. Participants were randomly assigned to either usual care alone or with the addition of acupuncture 5 days per week for 3 weeks. The primary outcome was death or disability at 6 months post-stroke, defined as a Barthel Index score of not more than 60 points.

5. The ICARE (Interdisciplinary Comprehensive Arm Rehabilitation Evaluation) trial recruited 361 patients between 16 and 106 days (mean of 46 days) after stroke at seven sites in 45 months ^11^. Participants were randomly assigned to usual and customary care, a 30-hour programme of task-oriented motor rehabilitation for the upper limb delivered over 10 weeks, or dose-equivalent usual and customary upper-limb therapy. The primary outcome was the change in the log-transformed time score from the Wolf Motor Function Test, between baseline and 12 months after randomisation.

6. The SWIFT (Soft-Scotch Walking Initial FooT) Cast trial recruited 105 patients between 3 and 42 days (mean of 21 days) after stroke at two sites in 25 months. Participants were randomly assigned to conventional physical therapy with a conventional ankle-foot orthosis or with a customised ankle-foot orthosis ^12^. The primary outcome was walking speed at the end of the 6-week intervention.

None of these trials found that the intervention was superior to standard care, and one found that the intervention worsened outcomes ^9^. These are disappointing results for many of the clinicians and researchers who worked on the trials, for the funding bodies, and most importantly for the patients involved and the wider stroke community. Although these trials at least demonstrate that large multi-centre rehabilitation trials can be conducted at the subacute stage of stroke, it should also be noted that two of the larger trials took several years to complete, recruited less than 10% of screened patients, and had recruitment rates less than one patient per month per site ^9, 11^. A low proportion of patients recruited raises potential concerns about the generalisability of the intervention, whereas a slow recruitment rate raises potential concerns about the feasibility of similar studies in the future.

Low and slow recruitment can be the product of strict inclusion/exclusion criteria, typical of explanatory trials designed to show that a standardised treatment is efficacious in a carefully selected group of patients. Although these are features of a well-designed study, they can also limit the trial’s usefulness to real-world clinical practice. If the treatment is found to be beneficial, the clinician does not know whether to use it for a patient who would have been excluded from the trial or in a setting that cannot provide the precisely defined treatment.

The issue of generalisability has long been recognised ^13^ and needs to be explicitly addressed in stroke rehabilitation research. As a simple starting point, an intervention’s generalisability could be more easily appreciated if trials reported the proportion of patients for whom an intervention is suitable, even if they cannot participate in research because of factors such as reduced capacity for consent, having contraindications to research measures such as magnetic resonance imaging, or being enrolled in another study. This has been reported by two rehabilitation RCTs that recruited all patients within 30 days of stroke. One reported that the intervention was suitable for 40% of all admitted stroke patients and 9% were eligible for participation in the trial ^14^. The other reported that the intervention was suitable for 11% of all admitted stroke patients and 3% were eligible for participation in the trial ^15^. Distinguishing between eligibility for the intervention and eligibility for research provides a clearer picture of the proportion of patients who could potentially benefit from the intervention if it were part of routine clinical practice.

## What might need to change in order to achieve a breakthrough in stroke rehabilitation?

Two reports published in March addressed this important question. The first, from Juka Jolkkonen and Gert Kwakkel, made several useful recommendations ^16^. These include standardisation of terminology for recovery, greater treatment contrast between the experimental and control arms of trials, clearer definition of ‘usual and standard care’ when it is the control condition, and the use of outcome measures that can distinguish between true neurological recovery and adaptation or compensation strategies. These authors also identify low sample size as a major problem for most stroke rehabilitation trials ^16^, although solving this problem alone with large multi-centre trials is unhelpful if other aspects of trial design are not also improved.

The second report, from Julie Bernhardt and colleagues on behalf of the Stroke Recovery and Rehabilitation Roundtable ^17^, clearly identifies many limitations in current stroke rehabilitation research and signals broad agreement that these must be overcome in order to advance rehabilitation research. Some of the limitations noted are that the theoretical or mechanistic basis of interventions is often not well articulated, the dose of the intervention can seem arbitrary and poorly controlled, and the timing and type of outcome measures used are highly variable between trials. The report advocates the use of more clinically relevant animal models, agreed biomarkers for patient stratification, the systematic reporting of interventions and their fidelity, and the use of a core set of outcome measures made at agreed time points.

What about the interventions themselves? Those tested in the six studies summarised above were variations on current practice, possibly because these are easiest to evaluate within the clinical setting, using existing staff and resources. Even the large, well-designed and carefully controlled studies outlined above were unable to detect a difference between standard care and a variation of standard care in the ‘noisy’ environment of SBR. This may be unsurprising given recent evidence that current therapy practice does not seem to interact with SBR ^18, 19^. Greater contrast is needed ^16^, as well as the evaluation of novel treatments that bear little resemblance to current therapy practice and directly target the neurobiological processes responsible for recovery ^5^. This might require ‘aspirational’ trials ^20^ of interventions that could not be delivered with current rehabilitation resources and service delivery models.

Assuming there are interventions that can meaningfully enhance recovery during the initial weeks after stroke, how might these eventually translate to widespread clinical practice? A further consideration, not explicitly made by recent reports ^16, 17^, is that translation requires a more pragmatic trial design. Pragmatic trials are designed and reported in a way that helps clinicians evaluate how and with whom the intervention could be used in clinical practice ^13^. They often take place in a usual care setting, with minimal patient selection, and with a level of flexibility in delivery of the treatment that would be found if it were part of usual practice ^13, 20^. However, this runs counter in some respects to the greater levels of standardisation advocated for in recent reports ^16, 17^. Highly standardised protocols are an important component of explanatory trials, which aim to detect treatment efficacy under ideal conditions that minimise the messiness of real-world clinical practice. Researchers could aim at the design stage to more consciously position their trials on the explanatory-pragmatic continuum by using tools such as PRECIS (Pragmatic-Explanatory Continuum Indicatory Summary) ^21^. Trials at both ends of the continuum are needed and can be equally rigorous; they are simply designed to answer two different questions ^22^. Finally, a novel rehabilitation intervention is highly unlikely to work for everyone, and it does not need to. But it is more likely to become part of clinical practice if trials are designed and reported ^23^ in a way that allows clinicians to judge which patients are most likely to benefit from it in the real-world rehabilitation setting.

## Conclusions

The neutral outcomes of several large multi-centre rehabilitation trials conducted during spontaneous recovery from stroke ^7– 12^ give pause for thought. These recent trials represent steps in a useful direction, towards testing interventions at the time when most stroke rehabilitation takes place. They seem to have also prompted discussion about how best to advance stroke rehabilitation research through improved and harmonised trial design ^16, 17^. The need for greater contrast between experimental interventions and standard care, and the potential importance of enhancing SBR, are increasingly apparent. Interventions that are efficacious during spontaneous recovery also need to be trialled with a more pragmatic approach to evaluate their effectiveness and facilitate their translation to clinical practice. Clearly, we need to be doing something different in order to make a difference.

## Abbreviations

RCT, randomised controlled trial; SBR, spontaneous biological recovery.
